# The Passage of S100B from Brain to Blood Is Not Specifically Related to the Blood-Brain Barrier Integrity

**DOI:** 10.1155/2010/801295

**Published:** 2010-07-08

**Authors:** Andrea Kleindienst, Christian Schmidt, Hans Parsch, Irene Emtmann, Yu Xu, Michael Buchfelder

**Affiliations:** ^1^Department of Neurosurgery, University Erlangen-Nuremberg, Schwabachanlage 6, 91054 Erlangen, Germany; ^2^Institute of Laboratory Medicine, University Erlangen-Nuremberg, 91054 Erlangen, Germany

## Abstract

Following brain injury, S100B is released from damaged astrocytes but also yields repair mechanisms. We measured S100B in the cerebrospinal fluid (CSF) and serum (Cobas e411 electrochemiluminescence assay, Roche) longitudinally in a large cohort of patients treated with a ventricular drainage following traumatic brain injury (TBI) or subarachnoid hemorrhage (SAH). Statistical analysis was performed with SPSS software applying the Mann-Whitney rank sum test or chi-test where appropriate. S100B in CSF and serum was significantly increased following TBI (*n* = 71) and SAH (*n* = 185) for at least one week following injury. High S100B levels in CSF and serum were inconsistent associated with outcome. The passage of S100B from CSF to blood (100^∗^serum_S100B_/CSF_S100B_) was significantly decreased although the albumin quotient suggested an “open” blood-CSF barrier. Events possibly interfering with the BBB did not affect the S100B passage (*P* = .591). In conclusion, we could not confirm S100B measurements to reliably predict outcome, and a compromised blood-CSF barrier did not affect the passage of S100B from CSF to serum.

## 1. Introduction

There is a desire for a reliable indicator to accurately determine the extent of brain injury and consequent prognosis. The measurement of putative biochemical markers, such as the S100B protein, has been proposed in this role. Furthermore, such a biomarker would aid in identifying events contributing to secondary brain damage and monitoring the success of therapeutic interventions.

Over the past decade, numerous studies have reported a positive correlation between S100B levels in blood or cerebrospinal fluid (CSF) and impaired neurological function following traumatic brain injury (TBI) [1], intracerebral hemorrhage [2], stroke [3], perinatal brain damage [4], septic encephalopathy [5], bacterial meningitis [5], or even in major depression [6], and extracranial injuries [7]. Furthermore, S100B levels have been used to monitor therapeutic effects such as the application of hypertonic saline in TBI [8] or of naloxone in epilepsia [9]. However, considerable evidence indicates that S100B is not only a biomarker of brain damage but also represents ongoing neuroregeneration [10]. Moreover, contradictory data interpretation exists with regard to the contribution of an altered blood-brain barrier (BBB) to S100B serum levels [11, 12].

Although in cell cultures the injury-induced S100B release continues to increase up to 48 hours [13, 14], in patients S100B serum levels have been reported to be highest directly after the injury and become normalized within 24 hours in a high percentage of cases, even in those patients with a bad outcome [15]. The underlying mechanism describing the passage of S100B from brain to blood following acute brain injury has not yet been clarified, nor does an unequivocal data interpretation exist regarding cerebral S100B levels and their correlation to serum S100B levels.

Opposite to the BBB, the compartmental barrier within the ventricles is not at the level of the blood vessels but between the epithelial cells that form the inner CSF-facing surface of the choroid plexus. Since the choroid plexus are of mesodermal origin, their leaky capillaries are one of the exceptions to the rule that almost all capillaries in the central nervous system (CNS) form tight junctions between their endothelial cells thereby establishing the BBB. CSF is directly secreted by the choroid plexus into the ventricles constituting a blood ultrafiltrate and is also derived from the extracellular fluid (ECF). The ECF communicates reasonably freely with the ventricular CSF through normal nonbarrier spaces between ependymal cells [16]. CSF production is measured by dilution studies, and a total volume of 130 ml CSF in men is renewed every 5–7 hours. The classic view of CSF removal is to pass through the arachnoid villi into the venous sinuses by bulk flow [17], but alternatively CSF may pass into the blood vessels driven by a mixing or pulsatile flow [18]. 

Although the functional assessment of the dynamics of protein passage from blood to brain or *vice versa* in CNS disorders is of general interest, in patients the means of quantification are limited. Since blood and CSF are readily accessible, calculating respective ratios is reasonable. The albumin_CSF_/albumin_serum_ quotient (Q_A_) has been established as the “golden standard” for the assessment of blood-CSF barrier dysfunction [19, 20] although it is occasionally mistaken to measure BBB permeability. The 66 KD protein albumin is synthesized peripherally, is not catabolised within the CNS, and does not readily diffuse across an intact BBB. In adults, normal values are defined as a Q_A_ ≤ 0.007, and a damaged or open blood-CSF barrier is defined as mild (Q_A_ = 0.007–0.01), moderate (Q_A_ = 0.01–0.02), and severe (Q_A_ ≥ 0.02), respectively [21]. Applying the Q_A_, it is important to note that the ratio has been established in lumbar CSF. Extensive studies on the dynamics of blood- and brain-derived proteins across the blood-CSF barrier support the view that blood-CSF barrier dysfunction is a biophysical concept of increased molecular flux with decreasing CSF flow rate rather than a morphological “leakage” model [22]. 

Thus, interpreting the serum and CSF levels of the 22 kD dimeric neurotrophic protein S100B following acute brain damage is a dual challenge. Firstly, reasonable evidence exists that S100B is not only passively released by damaged astrocytes but also actively secreted and acts in a positive paracrine manner to foster neuronal repair or regeneration. Secondly, protein reabsorption accompanying CSF turnover emerges as a possibility. Facing the limitations of in vitro and in vivo experimental models, we confined ourselves to data collection in the clinical setting of acute brain injury. In particular, the purpose of the present study was (i) to examine the temporal profile of S100B release into CSF and blood in a large cohort of patients following TBI and subarachnoid hemorrhage (SAH) longitudinally, (ii) to calculate the ratio of S100B in the CSF/serum in order to estimate the passage from CSF to blood, and (iii) to correlate the respective S100B levels with the neurological function and recovery as well as with specific events known to interfere with the BBB integrity.

## 2. Methods

### 2.1. Subjects

The study was approved by the Ethics Committee of our hospital and was conducted in compliance with the Declaration of Helsinki. Informed consent was obtained from the next of kin of the patient. Patients were included unless one of the following exclusion criteria was present: age <18 years; pregnancy or nursing state; primary central nervous disorders (e.g., meningitis, neoplasm, or known epilepsy); expected to die within the first 48 hours; melanoma; severe burns, orthopaedic surgery, or cardiac bypass surgery. Patients were enrolled into two groups, one presenting with isolated TBI requiring ventriculostomy and catheter placement (*n* = 71) and the other presenting with SAH (*n* = 185). Normal values of S100B in serum and CSF in healthy controls had been established in the past in [12]. Since the normal values in this study were established in the lumbar CSF of control patients, we applied a 3.5 correction factor according to the CSF flow rate model of Reiber [22].

In the study patients, ventriculostomy catheters were placed as part of the clinical care within 12 hours of admission. Ventriculostomy catheters are typically placed to monitor intracerebral pressure in patients with severe TBI (Glasgow Coma Scale [GCS] score ≤8) and an intracranial injury on computed tomography (CT) scan. However, these catheters are also placed in those with severe TBI and a normal CT if two or more of the following factors are present: age >40 years, unilateral/bilateral motor posturing, and systolic blood pressure <90 mmHg [23]. In some cases, subjects with initial GCS scores above 8 received ventriculostomy catheters because of subsequent clinical deterioration and were included in the study. Subjects were eligible for inclusion as a SAH subject if the diagnosis was confirmed by CT or an abnormal lumbar puncture. 

Extracranial and brain injury were documented by CT, neurological function by the GCS and Glasgow outcome Score (GOS), as well as intensive care scores (APACHE and/or SAPS). All events potentially interfering with S100B passage were recorded: hypotonia (mean blood pressure < 65 mmHg), hypoxia (SpO2  < 90%), hyperthermia (>38°C), increased intracranial pressure (ICP > 20 mmHg), treatment with mannitol, increased cerebral blood flow velocity (>80 cm/s), and treatment modalities as aneurysm surgery or coiling, change of ventricular drainage, shunt implantation, or tracheotomy.

### 2.2. Sample Collection and Processing

In TBI and SAH subjects, we collected blood and CSF samples daily at 8 AM for up to 4 weeks postinjury. For all blood draws, 4 ml of venous or arterial blood were drawn into serum separator tubes and centrifuged at 3000 rpm for 10 minutes at room temperature. The cellular components were discarded and the serum stored at −80°C until used for assays. CSF was collected at the time of blood draws. For each CSF sample, 5–10 ml were collected into a 15 ml polypropylene tube, immediately placed on ice, and centrifuged at 3000 rpm for 10 minutes at room temperature. The cellular components were discarded and the remaining sample stored at −80°C until used for assays. Serum S100B concentrations were measured with the Cobas e411 S100 electrochemiluminescence assay, Roche Diagnostics. S100B concentrations in the serum samples were determined using the standard curve generated from the absorbance of the standards. The lower detection limit of this assay is 0.005 *μ*g/l, the upper limit 39 *μ*g/l. In order to quantify the S100B passage from CSF into blood, we calculated the S100B ratio in serum originating from CSF ( = 100*serum_S100B_/CSF_S100B_).

Albumin was analysed by immunochemical nephelometry, BN Prospec, Siemens Diagnostics, as described elsewhere [24]. The standard calibration curve for CSF measurements was also applied for serum measurements following an instrumented dilution either by x400 or x2000. Analysis of the blood-CSF barrier function was determined using the CSF_albumin_/serum_albumin_ ratio (Q_A_). Daily albumin values were measured in CSF and serum, and the Q_A_ was calculated. Since our albumin analyses were performed in ventricular CSF, a correction factor for a ventricular to lumbar CSF gradient of 1 : 2.5 was applied, and a relevant disturbance of the blood-CSF barrier function was assumed if the Q_A_ exceeded 0.0028 [21, 22].

### 2.3. Statistical Analysis

All values are given as mean ± SEM. The statistical analysis was performed using SPSS (SPSS Inc., Chicago, IL) and R (open source software). Group comparisons, such as TBI versus SAH, or good (GOS 4 + 5), moderate (GOS 3), and worse (GOS 1 + 2) outcome were made applying the Mann-Whitney rank sum test since no normal distribution was present. Correlation analysis between S100B values and the Q_A_ was performed using the nonparametric Kendall's tau_b Pearson correlation test. The chi-test was used to compare the incidence of events associated with an increase or decrease of S100B levels. Significance was defined as *P* < .05.

## 3. Results

**Figure 1:**
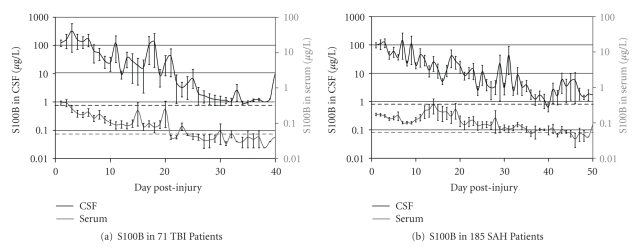
Mean S100B levels in CSF (a) and serum (b) following subarachnoid hemorrhage (SAH) or traumatic brain injury (TBI). The values are given as mean ± SEM.

We included 71 patients admitted for TBI and 185 patients following SAH. The normal S100B values had been established in control patients undergoing pituitary surgery treated with a lumbar drainage at 0.07 *μ*g/l in serum and at 2.87 *μ*g/l in CSF applying a 3.5 correction factor to normalize for the negative ventricular-lumbar CSF gradient of brain-derived proteins. S100B in CSF was significantly increased up to day 7 following TBI (64.98 ± 272.39 *μ*g/l, *P* = .024) and SAH (146.90 ± 1374.86 *μ*g/l, *P* = .009) and in serum up to day 8 following TBI (0.16 ± 0.30 *μ*g/l, *P* = .032) and up to day 14 following SAH (0.33 ± 1.12 *μ*g/l, *P* = .016). The time course of the S100B concentration in serum and CSF in 185 SAH patients and 71 TBI patients is displayed in Figures 1(a) and 1(b). Statistical comparison between the different types of acute brain injury by the Whitney-Mann rank sum test revealed that S100B in CSF and serum was significantly higher following TBI than SAH for the first 5 days (*P* < .05). 

### 3.1. S100B Serum/CSF Ratio and the Blood-CSF Barrier

In order to quantify the S100B passage from CSF into blood, we compared the ratio serum_S100B_/CSF_S100B_. In the control patients, S100B in serum comprised around 2.8% of the respective CSF concentration, applying a 3.5 correction factor for the negative ventricular to lumbar CSF gradient. The S100B ratio in SAH and TBI patients was significantly reduced for the first 4 days (*P* < .05). In accordance to the literature, we quantified the ratio QA, respectively, CSF_Albumin_/serum_Albumin_ reflecting the passage of albumin from blood to the brain. The Q_A_ was significantly increased over the investigation period (day 1: 0.015 ± 0.012, day 10: 0.016 ± 0.013) as compared to normal values (Q_A_[normal for ventricular CSF] ≤ 0.0028, *P* < .05). There was no correlation between the S100B ratio and the Q_A_ (day 1: *r* = 0.233, *P* = .615, day 7: *r* = 0.110, *P* = .860).

### 3.2. Prediction of Outcome

Within the groups, there was no consistent correlation between S100B concentrations in either serum or CSF and neurological function as assessed by GCS. The statistical analysis of outcome prediction and S100B levels revealed inconsistent findings, especially in TBI patients. SAH patients with worse outcome (GOS 1 + 2) had significantly higher S100B serum levels on day 2 (*P* = .042), day 3 (*P* = .042), and day 4 (*P* = .031) as compared to moderate outcome (GOS 3) and on day 5 (*P* = .006), day 7 (*P* = .004), day 11 (*P* = .012), day 12 (*P* = .008), day 13 (*P* = .003), day 14 (*P* = .003), day 15 (*P* = .032), and day 17 (*P* = .036) as compared to good outcome (GOS 4 + 5). SAH patients with worse outcome had significantly higher S100B CSF levels on day 1 (*P* = .011), day 2 (*P* = .010), and day 3 (*P* = .010) as compared to moderate outcome and on day 5 (*P* = .011), day 7 (*P* = .021), day 10 (*P* = .003), day 12 (*P* = .012), and day 13 (*P* = .028) as compared to good outcome. SAH patients with worse outcome had a significantly impaired S100B CSF/serum passage on day 3 (*P* = .026) as compared to moderate outcome and on day 10 (*P* = .039) as compared to good outcome. TBI patients with worse outcome had significantly higher S100B serum levels on day 2 (*P* = .019) as compared to good outcome. TBI patients with worse outcome had significantly *lower* S100B CSF levels on day 3 (*P* = .016) as compared to moderate outcome. TBI patients with worse outcome had a significantly *improved* S100B CSF/serum passage on day 3 (*P* = .016) as compared to moderate outcome.

### 3.3. Events Affecting S100B Levels

**Figure 2:**
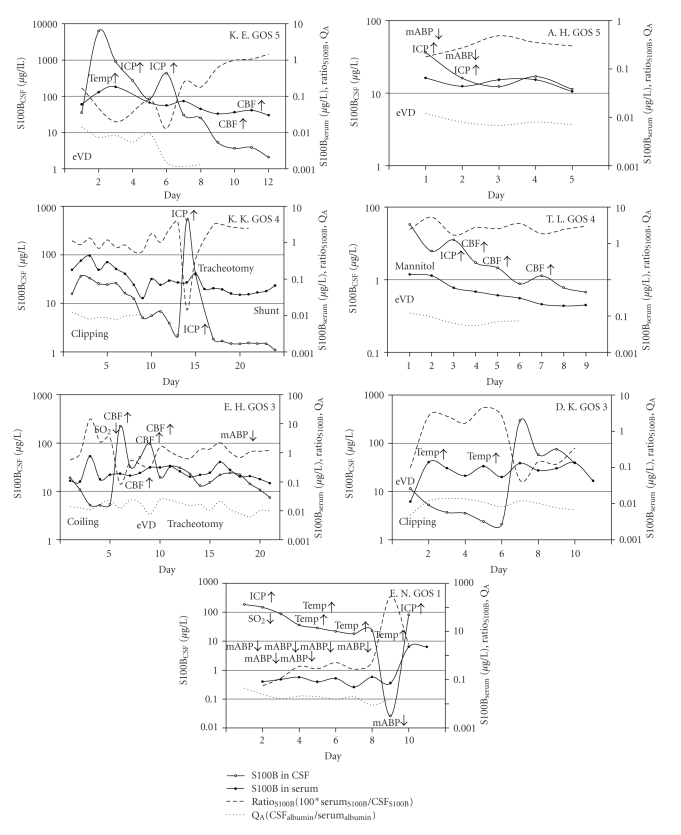
For individual patients, S100B in serum and CSF as well as the ratio serum_S100B_/CSF_S100B_ and the Q_A_CSF_Albumin_/serum_Albumin_ are displayed as well as events affecting the blood-brain barrier or blood-CSF barrier integrity. Note that the values are displayed on a logarithmic scale. GOS, Glasgow Outcome Score.

**Figure 3:**
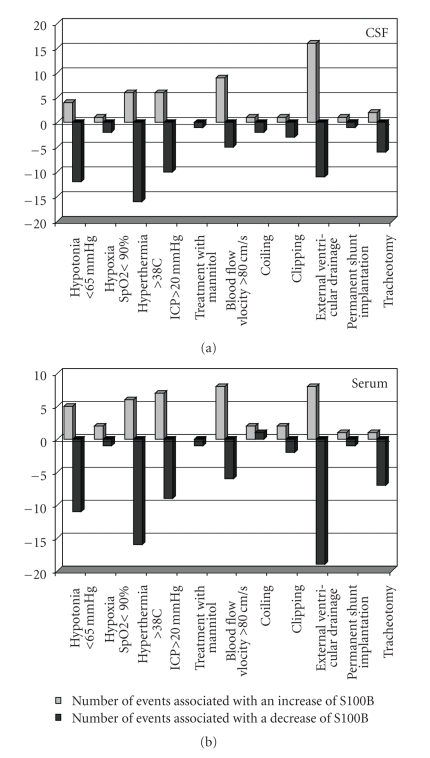
Events potentially interfering with the blood-brain barrier (BBB) and the effect on the S100B concentration in CSF and serum following subarachnoid hemorrhage (SAH) or traumatic brain injury (TBI). The chi-test did not reveal any impact of events on S100B levels.

In 30 patients, we analyzed events possibly interfering with the BBB in detail (Figure 2). Neither hypotonia (mean blood pressure < 65 mmHg) nor hypoxia (SpO2 < 90%), hyperthermia (>38°C), increased intracranial pressure (ICP > 20 mmHg), treatment with mannitol, increased cerebral blood flow velocity (>80 cm/s), or treatment modalities as aneurysm surgery or coiling, change of ventricular drainage, shunt implantation or tracheotomy did affect the passage of S100B or albumin through the blood-CSF barrier (*P* = .591). The incidence of events is displayed in Figure 3.

### 3.4. Contribution of Extracerebral S100B Sources

In TBI patients, the contribution of extracerebral sources to S100B levels was assessed. One patient suffered from a femur fracture contributing to excessively elevated S100B serum levels on admission (1.22 *μ*g/l). However, S100B was cleared from serum on the following day (0.25 *μ*g/l), and CSF levels on admission remained below mean values (36.98 *μ*g/l), and for the total investigation period. In two other patients with a fracture of the clavicle, S100B serum levels were not affected. Furthermore, S100B is expressed in relevant concentrations in adipose tissue [25], and S100B serum levels were reported to correlate with the BMI and speculated to be closely linked to an altered energy metabolism in diabetic patients [26]. In our study, obesity did not significantly affect S100B serum levels while in diabetic patients, S100B serum levels were significantly higher than in nondiabetic ones (*P* = .013).

## 4. Discussion

To the best of our knowledge, this is the first study to investigate concurrent concentrations of S100B in serum and CSF in more than 250 patients following acute brain injury longitudinally for up to 4 weeks. As it has been iterated by a plethora of authors, we found some correlation of high S100B levels and worse outcome, but far from any prerequisite of unequivocal outcome prediction. Opposite to common reasoning, in our study the passage of S100B from CSF to serum was impaired following acute brain injury. We could not confirm any contribution of a compromised blood-CSF barrier to S100B serum levels. 

### 4.1. Release of S100B into CSF

Neither the role of the glial protein S100B in the acutely injured brain nor the release into the ECF and the subsequent passage to the CSF and blood has been established. From *in vitro* injury, we learned that S100B is released into the culture medium [13, 14]. The ECF communicates with the CSF through normal nonbarrier spaces [16], presumably allowing S100B to pass freely from the ECF to the CSF. Thus, it is reasonable to assume that S100B CSF concentration measured following human brain injury reflects the S100B release into the ECF. The injury-induced S100B release in cell cultures displayed an upward slope over the investigation period [14] demonstrating an active stimulated release contributing to the total S100B concentration measured. *In vivo* data suggest a S100B release due to learning and memory processes [27, 28]. In patients, electroshock therapy did not affect S100B serum levels [29], but acute psychosis resulted in an increased concentration of S100B in CSF and serum [30]. The long-lasting increased S100B levels in CSF found in our patients following SAH or TBI are unlikely to result purely from brain injury. Furthermore, we were unable to verify any consistent correlation of S100B CSF levels and injury severity or outcome. However, since a strong S100B immunopositivity of the ependymal and choroid plexus epithelia has been observed although the functional consequences have not been elucidated yet, we cannot exclude a contribution from these cells to the S100B CSF concentration [31]. A limitation of our study is comparing the ventricular CSF measurements from our patients with lumbar CSF measurements from controls subjects of a previous study. To eliminate any interference of ventricular-lumbar protein gradients, we applied an estimated “correction factor” of 3.5 for S100B and of 0.4 for albumin [22]. Taken together, the literature and our findings imply an active stimulated S100B release into CSF reflecting neuronal-glial activation, synaptic plasticity, or neuroregeneration rather than to result from injured cells.

### 4.2. Brain-CSF-Blood Barrier

Increased S100B concentrations in the blood have been attributed to the passage through an impaired BBB following brain injury [26], whenever an extracerebral origin of S100B was excluded [26, 32, 33]. However, contradictory data interpretation exists with regard to the contribution of an altered BBB to S100B serum levels [12]. 

The BBB prevents diffusion of most water-soluble molecules over 500 Da. Although the albumin_CSF_/albumin_serum_ quotient (Q_A_) has originally been described for the blood-CSF barrier dysfunction [19, 20], several authors calculated indices using the Q_A_ as a measure of “BBB” permeability following TBI, such for the intercellular adhesion molecule-1 (ICAM-1) [34], for the antiinflammatory transforming growth factor-beta (TGF-beta) [35], or for the complement-derived soluble membrane attack complex (sC5b-9) [36], and they reported the respective CSF levels paralleling the “BBB” function as assessed by the Q_A_. However, other authors could not confirm such a correlation, for example, assessing the cerebral production of interleukin (IL)-10 [37] or IL-6, IL-8 and IL-10 [38]. 

The lessons we learned from these controversies are twofold. First, any ratio CSF_SUBSTANCE_/serum_SUBSTANCE_ does reflect the passage of the respective protein through the blood-CSF barrier that is clearly to distinguish from the BBB and is a measure of an altered CSF flow [22]. Second, the albumin_CSF_/albumin_serum_ quotient Q_A_ allows inference on proteins around 66 KD, while the dimeric 22 KD protein S100B may comply different dynamics.

### 4.3. Passage of S100B from Brain to Blood

Following a preliminary study including few patients and assessing the release and wash-out pattern of S100B in serum and CSF, the importance to know the underlying pathology and timing in interpreting S100B levels has been highlighted [39]. We analyzed the impact of several pathophysiological dysregulations like hypotonia, hypoxia, hyperthermia, increased intracranial pressure, vasospasm, and craniotomy known to affect the BBB. Furthermore, the BBB has been reported to be opened osmotically [40]. In our study, those events likely to interfere with the BBB and treatment with mannitol did not affect the passage of S100B from the CSF to blood. 

Opposite to the BBB, the compartmental barriers between the CSF and blood are leaky capillaries of the choroid plexus allowing protein secretion into the CSF. Little is known whether this secretion is unidirectional or may allow the reabsorption of proteins from the CSF into blood. Furthermore, the relevance of the blood-CSF barrier in acute brain damage remains unclear. Ultrastructural examinations of the choroidal epithelial cells forming the CSF-blood barrier following experimental injury demonstrate pronounced changes lasting up to 4 weeks postinjury [41]. Accordingly, the reduced ratio of S100B serum/CSF found in our patients may result from damaged choroidal epithelial cells hampering with the S100B passage from CSF to blood [31]. However, considerable evidence indicates that S100B is not only a biomarker of brain damage but also represents ongoing repair or neuroregeneration [10]. Thus, the reduced passage of the neurotrophic protein S00B from CSF to blood may result from an increased demand in injured tissue.

## 5. Conclusion

Although there is a reasonable desire for a reliable indicator to accurately determine the extent of brain injury and to monitor therapeutic interventions, advocating S100B in this role remains problematic. While a substantial body of evidence demonstrates an association between S100B and bad outcome after brain injury, it is important to be aware that proof of an association is not proof of causation in science. In the present large cohort of patients, the concurrent measurement of S100B in serum and CSF, we found some association of high S100B levels and worse outcome, but far from any prerequisite of unequivocal outcome prediction. Opposite to common reasoning, we found the passage of S100B from CSF to serum impaired following acute brain injury. We could not confirm any contribution of a compromised BBB or blood-CSF barrier to S100B serum levels.
